# Evaluation of masticatory efficiency and OHRQoL in implant-retained overdenture with different numbers of implant in the edentulous mandible: a one-year follow-up prospective study

**DOI:** 10.1186/s40729-024-00519-0

**Published:** 2024-03-14

**Authors:** Kawe Sagheb, Stefan Wentaschek, Monika Bjelopavlovic, Manfred Berres, Leonardo Díaz, Shengchi Fan, Eik Schiegnitz, Bilal Al-Nawas, Keyvan Sagheb

**Affiliations:** 1 Department of Prosthetic, University Medical Center Mainz, Mainz, Germany; 2 Institute for Medical Biostatistics, Epidemiology and Informatics (IMBEI), University Medical Center Mainz, Mainz, Germany; 3 Postgraduate School, Faculty of Dentistry, Universidad de Chile, Santiago, Chile; 4 Department of Oral and Maxillofacial Surgery-Plastic Operations, University Medical Center Mainz, Mainz, Germany

## Abstract

**Purpose:**

The aim of this article is to evaluate to the masticatory function performance and Oral Health-related Quality of Life (OHRQoL) in implant-retained overdenture compared with different implant number placements in the edentulous mandible.

**Methods:**

From 2013 to 2015, each patients received 3 implants (iSy-Implant, Camlog, Wimsheim, Germany) in intraforaminal mandible (34, 41/31, 44). After operation, inserted implants were gradually loaded and incorporated into an overdenture with a self-aligning attachment system (Locator abutments) in 3 + 3 + 3 months. Five checked points were performed chewing cycle test with multicolored chewing gum and OHIP-G14 questionnaire and a sum score questionnaire as following: pre-operation, one implant load (41/31), two implants loaded (33,43), three implants loaded and 1-year follow up.

**Result:**

A total of 10 patients with 30 implants were placed, the survival rate of the implants was 100% within 1-year follow-up. Regarding the masticatory function analysis, for the higher number of chewing cycles, the higher mixing rate was observed. After 1 year, the inter-mixing rate without significant changes was found compared to the time after three implants were loaded with attachment system. The mean value of OHIP-G14 was 30.4 preoperatively, 21.1 after loading the first locator, 10.7 after loading two locator abutments, and 3.2 after loading all three locator abutments. After 1 year, OHIP-G14 was 2.6 without significantly changed. The mean of the sum score was 15.5 preoperatively, 27.8 after activation of the first locator, 39.4 after activation of two locators, 46.2 after activation of all three locators, and 47.3 after 1 year. An increase of 0.7 sum score units per time point was observed. No significance was detectable, analogous to OHIP-G14, compared to the time of activation of all three locator setups (*p*-value = 0.22).

**Conclusions:**

A significant improvement in masticatory function performance and OHRQoL was evaluated with the increasing number of implants with locator attachment in edentulous mandible. With the investigation of the OHIP-G14 and sum score, the results of patient report outcome might be associated with the increase in the number of implants.

**Graphical abstract:**

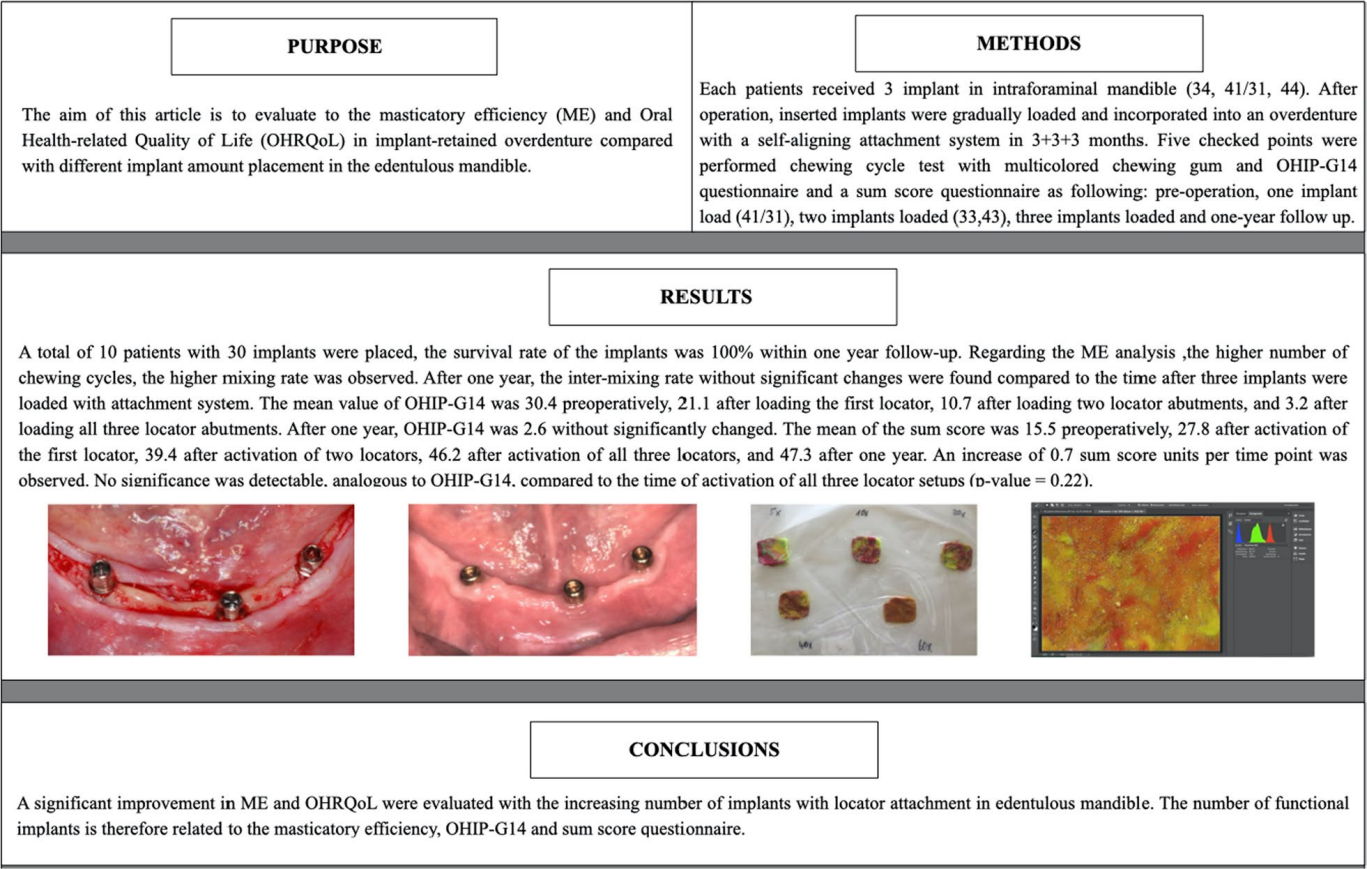

## Introduction

Edentulous jaws are often associated with masticatory dysfunction, which is usually accompanied by significant resorption of atrophic alveolar bone in the clinical observation [1]. Bone resorption in the mandible occurs four times faster than in the maxilla, particularly in the lateral region [2, 3]. Once a tooth is lost, residual ridge resorption is an unavoidable process. Over time, the stability and retention of overdenture will decrease, resulting in insufficient and unsatisfactory masticatory function (MF) for patients [4–7]. The loss of alveolar bone between the crest and the mental foramen in the posterior area also makes implant placement more challenging and complex.

The replacement of missing teeth through various forms of prosthodontic treatment was found to be the most significant protective factor against masticatory dysfunction [8]. Dental implant can effectively improve the function and comfort of retained overdenture, which is increasing both the patient's MF and quality of life [9]. The Oral Health Impact Profile (OHIP) is the most widely used international instrument for oral health-related quality of life (OHRQoL). The short version with 14 questions (OHIP-14) is the most widely used and very practical [10, 11]. However, a critical question still being debated in the literatures, what is the minimal number of implants needed to support an overdenture to achieve adequate MF and satisfactory of OHRQoL in the edentulous mandible?

According to the McGill Consensus Conference, the standard treatment for mandibular implant-supported overdenture is to place two implants in the inter-foraminal area as attachment fixation [12]. However, the use of four implants can significantly improve a patient's OHRQoL and MF when combined with a removable prosthesis compared to this standard treatment [13, 14]. In addition, some minimalist concepts were proposed using a single implant in the midline to support the prosthesis, which can provide sufficient and satisfactory rehabilitation [15].

From the clinical perspective, rehabilitation with multiple implants that supports the mandibular overdenture through locator abutments is highly effective [16]. The 2023 ITI Consensus Group 4 stated that oral function significantly improves in edentulous patients who undergo rehabilitation with mandibular implant overdentures [17]. The abutment provides sufficient stability through external and internal retention, and is also easy to manage oral hygiene for the patient. From a bio-mechanical aspect, one implant improves prosthesis retention by increasing resistance to dislodging forces, but rotation is unavoidable. With two implants, the potential rotation is reduced to one axis based on the cantilever of the prosthesis, but zero rotation is not able to achieved [16]. Only with three implants distributed triangularly, it is possible to improve stability and avoid rotation force. Therefore, the stability of a prosthesis retained by three implants can be classified as superior to that of a restoration with one or two implants [18].

However, up to date, no research has been able to prove that three implants retaining an overdenture is superior to one or two implants in a prospective clinical study, especially in terms of masticatory function and OHRQoL. The primary objective of the current prospective study is to assess the correlation between MF performance and the number of implants (1 vs. 2 vs. 3) used to retain overdentures in the edentulous mandible. The secondary objective is to explore the influence of varying implant numbers on dental patient-reported outcomes using two questionnaires.”

## Material and methods

### Patients

The present study is a prospective clinical study conducted between 2013 and 2015, with 10 patients from the Department of Oral and Maxillofacial Surgery in collaboration with the Department of Prosthodontics at the University of Mainz. The study was approved by the ethics committee of the Rhineland-Palatinate Medical Association on February 25, 2013 (No. 837.097.13;8782-F). Patients were selected based on the following inclusion criteria:

- edentulous mandibula with sufficient bone height and width for placing three regular-size implants,
- functional maxilla (dentition, partial removable or complete denture).

Exclusion criteria:

- lack of informed consent,
- age less than18 years,
- pregnancy,
- refuse prosthetic treatment in the center,
- local, systemic and surgical exclusion criteria [19].

**Fig. 1:**
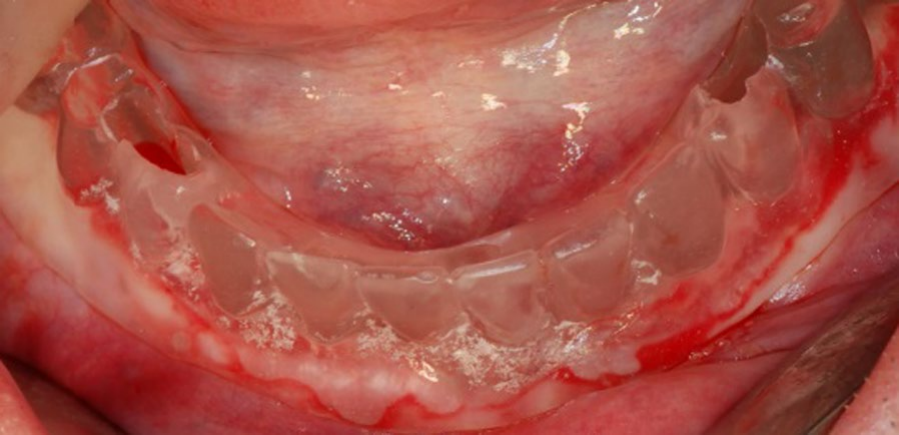
Three implants were placed with the guidance of radiographic guide

Enrolled patient declared their written informed consent to participate and an appointment was arranged for an examination. A masticatory efficiency test and questionnaires (OHIP-G14 and sum score) were performed in the examination. The degree of atrophic mandible was determined and documented based on clinical and radiographic examination. A new mandibular removable prosthesis was fabricated and tried-in; a radiographic template was made for implant installation as a surgical guide (Fig. 1).

All the patients received a total of 3 implants and follow-up with a standardized timeline. After 3 months of implants placement, locator titanium housing was gradually attached to the overdenture over a period of 9 months (3 + 3 + 3). Clinical follow-up was then conducted through an observational study of the patient cohort.

### Implant surgery protocol, follow-up and recall

**Fig. 2:**
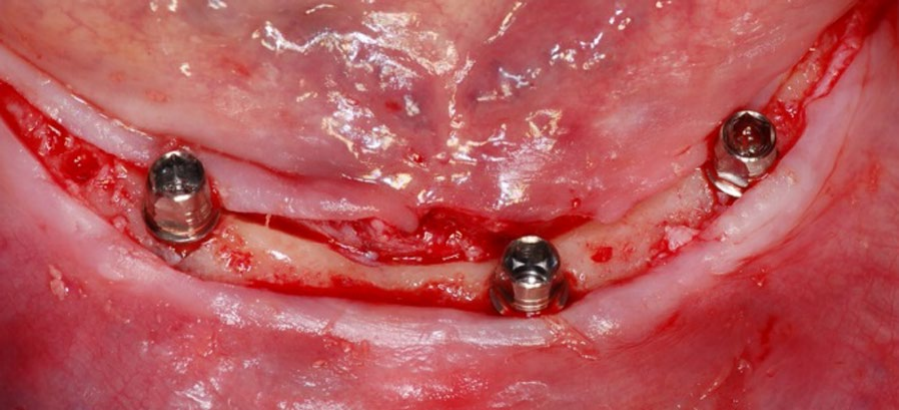
Implants were inserted in zones 34, 41/31, 44

Three dental implants (iSy-Implanta, Camlog, Wimsheim, Germany) were inserted in 34, 41/31, 44 under local anesthesia. After the crestal incision, the mental foramen were visualized bilaterally. With the aid of the surgical guide, implantation was performed according to the manufacturer's drilling protocol (Fig. 2). The final insertion torque was measured with the manual ratchet and resonance frequency analysis was performed after placement. An orthopantomography was taken after sutured and three transgingival abutments were installed. The prosthesis was hollowed out the corresponding tissue side for the space of healing abutments. After 10 days, the sutures were removed.

### Loading protocol and 1-year follow-up

**Fig. 3:**
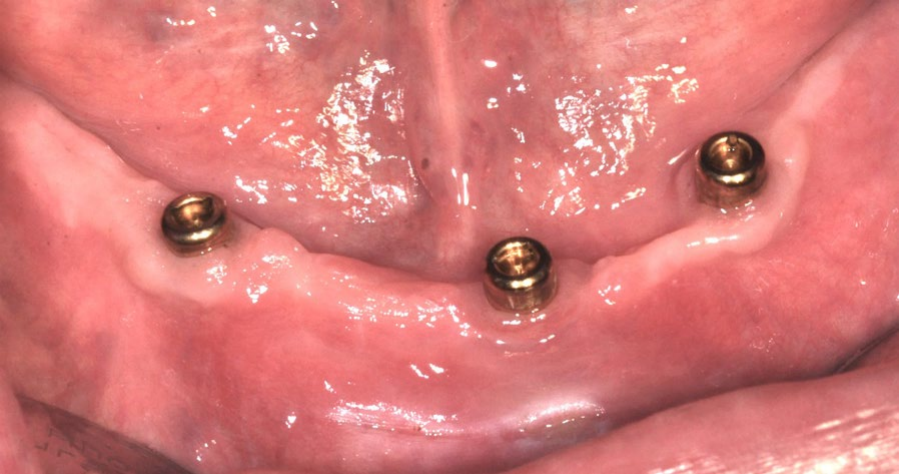
The final loading was made after 9 months of operation, all three abutments and components were placed on the implants

After 3 months of operation, three locator abutments were installed to the implants. Only the middle implant in the 31/41 area was incorporated to the prosthesis with titanium housing and retention rubber. After 6 months of operation, two implants at 34 and 44 were incorporated to the prosthesis with titanium housing and retention rubber, then the attachment components were removed from the middle implant and prosthesis. The final loading was made after 9 months of operation, all three abutments and components were placed on the implants and in the overdenture (Fig. 3).

In the each loading period, photos, masticatory efficiency test and questionnaires were taken in each patient. The last follow-up took place 1 year after 3 implants loading, and the masticatory efficiency test and questionnaires were performed again. And an orthopantomography was taken for routine radiological evaluation.

### Masticatory function test

**Fig. 4:**
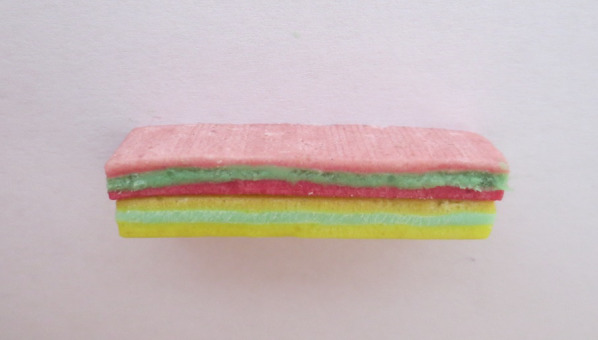
The ME test was performed using green and red color of chewing gum strips, which were placed on top of each other to create a testing chewing gum

**Fig. 5:**
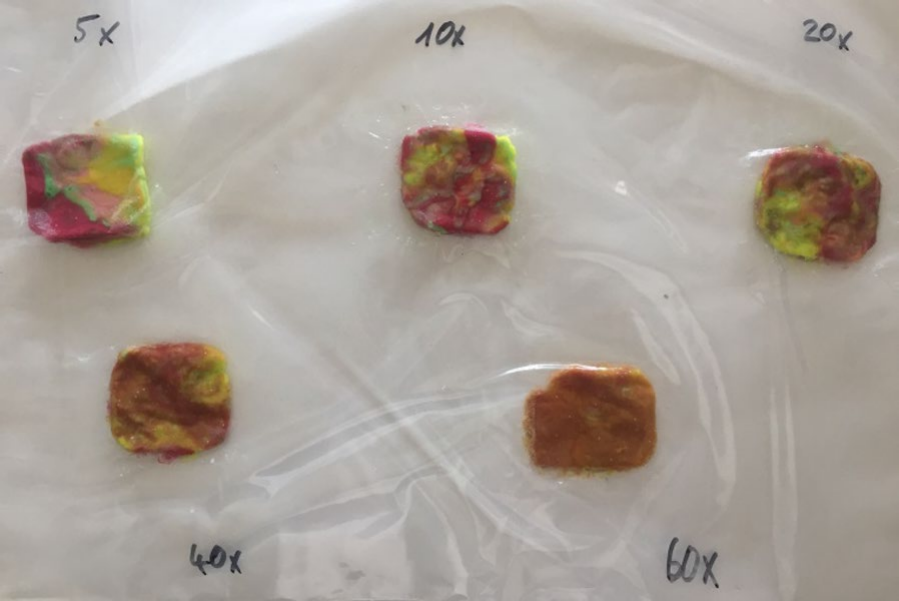
Tested chewing gum from one examination which chewed and shaped by plastic frame

To evaluate the MF from each loading period in each patient, it was determined that masticatory efficiency (ME) would be measured through a mixing test. The test was conducted five times on each patient at the following time points: before implant placement, after loading of the first locator abutment (31/41), after loading of the other two locator abutments (34,44), after loading of the three locator abutments (34,31/41,44), and during the 1-year follow-up examination (34,31/41,44). The ME test was performed using two different colors in green and red of chewing gum strips (Mentos Fruit 3) [20], which were placed on top of each other to create a testing chewing gum. Each testing chewing gum contained one red and one green gum placed on top of each other and lightly pressed together (Fig. 4). The patient was asked to perform 5, 10, 20, 40, and 60 masticatory cycles, and to apply maximum masticatory force. The chewing side did not play a role. Five different measurements were taken for 5, 10, 20, 40 and 60 chewing cycles (Fig. 5). A new testing gum was used for each measurement and 5 min rested between each examination.

**Fig. 6:**
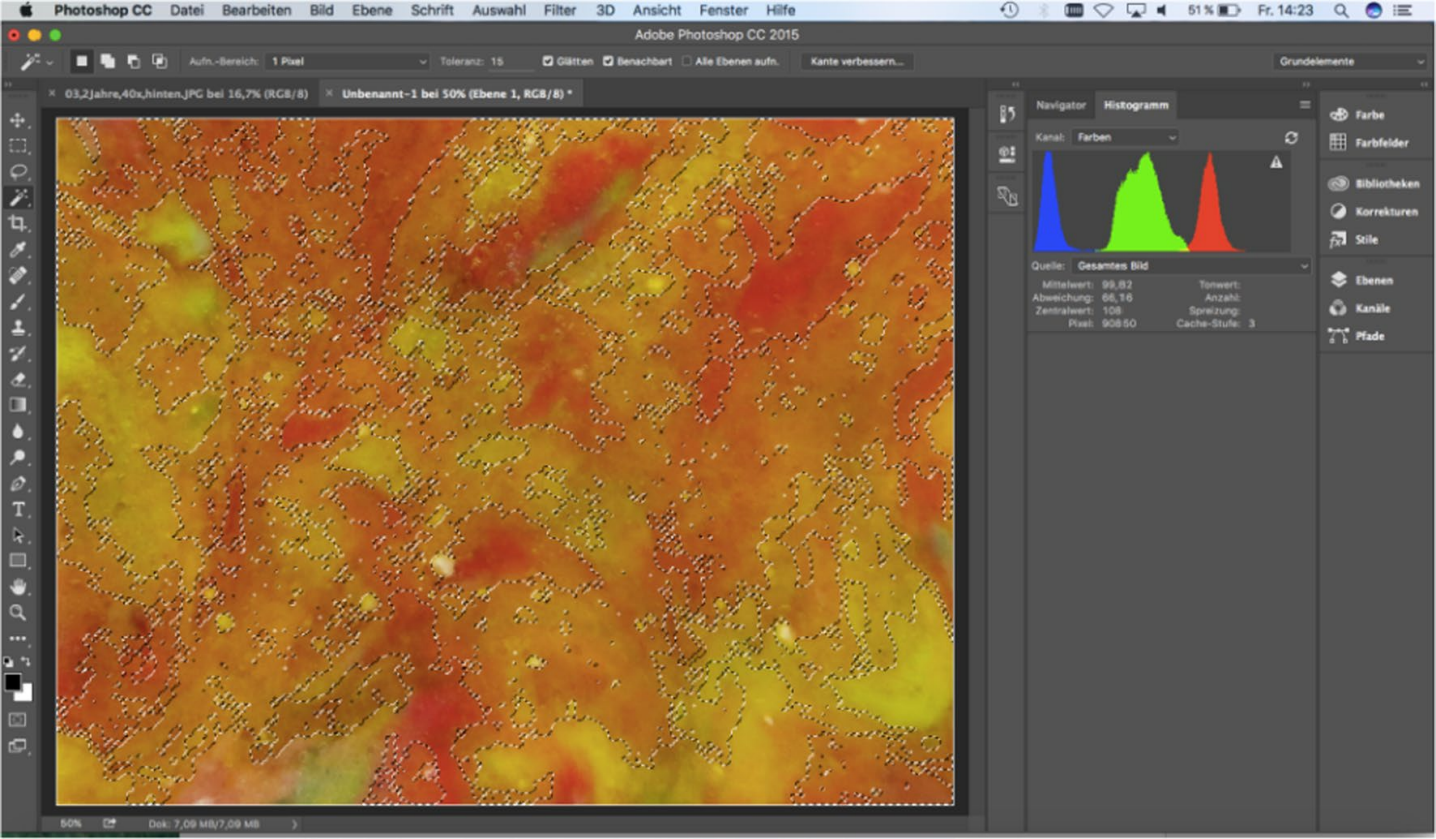
The photos were imported into Adobe Photoshop with the following settings applied to each image: a brightness value of 20 and a contrast value of 30. The histogram data included the total number of pixels, the mean value, the median value and the standard deviation within the section

The chewing gum was shaped into a rectangle of 40 × 32 mm with the aid of a plastic frame after the respective number of chewing cycles had been performed. The chewing gums were stored in a refrigerator at 7°C. Ten photographs were taken from both sides of each chewing gum with a Full HD camera (Canon IXUS 240 HS) to evaluate ME. A total of 50 photos from each patient were documented from the beginning to 1 year of follow-up. Each photo was evaluated using software (Adobe Photoshop CC 2015). First, a 15.24 × 11.65 cm rectangular section at a resolution of 118.11 pixels/cm was selected and saved as a new image from each photo. Then, the setup of 20 brightness value and 30 contrast value was applied to each image. After the setting, the histogram was called up via the path ImageHistogram. The histogram contained the following data: the total pixel, the mean value, the central value, and the standard deviation within the section (Fig. 6). These data were transferred to Excel. The evaluation of the image was done manually and individually for each image using the tool called "Magic Wand" to select the areas in each image with significantly changed colors from the original colors and mixed colors (green, pink, yellow, pink). These selected areas were set as intermixed areas of the chewing gum. In this step, the settings were as follows: tolerance value of the selection of 15 and activation of the options "Smooth" and "Adjacent". After that, a histogram was made via ImageHistogram. The histogram now indicated the pixel number of the selected blended area and its mean value, the central value, and the standard deviation within the area, as with the overall image section.

The blending rate of gum become apparent when the pixel count of the selected area was put in proportion to the total pixel count of the full photo crop. The percentage of color mixing rate, which was the average of the front and back of the test gum, was determined to be a measure of the ME of the particular patient. The higher the color mixing rate, the better the ME.

### OHIP-G14 and sum score

In the study, two different questionnaires were administered at each examination. The OHIP-G14 and the sum score questionnaire were given to the patients before implant placement, after placement of the first locator, after placement of the two and three locator abutments, and at the 1-year follow-up examination, respectively. The sum score questionnaire was designed to assess subjective masticatory performance and function. Patients were asked to rate their responses on a scale ranging from 1 (very poor) to 10 (very good) for the following five questions [21]:

"How satisfied are you with the prosthesis in your mouth?"

"How well can you bite off with your prosthesis?"

"How well can you speak with your prosthesis?"

"How well does your prosthesis fit?".

"How well can you eat solid food with your prosthesis?”

## Results

### The characteristics of the patients and implants

A total of 10 patients (8 female and 2 male) with a mean age of 65 ± 10.2 years (range 50 to 80) were included in the study. Seven patients were already completely edentulous in the maxilla and mandible, other three patients were partially edentulous in the maxilla and edentulous in the mandible. Nine patients presented Cawood and Howell V of atrophic mandible and one patient presented Cawood and Howell IV of atrophy [22]. None of them could place implant in the posterior area without vertical bone augmentation.

A total of 30 implants were installed with sufficient primary stability. The average final insertion torque during implant installation was 43 ± 4.8 Ncm. The average implant stability coefficients (ISQ) in zone 34, 31/41 and 44, measured through resonance frequency analysis, were 70.8 ± 3.9, 68 ± 3.7 and 69.5 ± 5.2, respectively. At 1-year follow-up, all implants were all functional, no failed was recorded. The survival rate of 30 implants was 100% in the 1-year control period.

### Masticatory function analysis

The higher the number of chewing cycles, the higher the mixing rate was observed (Table 1).

**Table 1:** Median values of mixing rates (%) with 5, 10, 10, 20, 40 and 60 chewing cycles preoperatively, after activation of the first, second, third locator and after 1 year of follow-up

Check-point	Pre-Op (%)	1 Imp-3 M (%)	2 Imp-6 M (%)	3 Imp-9 M (%)	3 Imp-1 year (%)
5 cycles	1.25	2.03	2.29	2.79	2.98
10 cycles	2.03	3.54	4.61	6.03	6.72
20 cycles	4.61	8.61	10.75	13.63	14.87
40 cycles	11.78	19.57	26.16	37.75	37.28
60 cycles	26.27	37.60	49.04	72.48	69.57

Imp: Implant

M: Months

An approximate linear increase in mixing with different slopes was observed from the initial state until after the third implant was incorporated. After 1 year, the intermixing without significant changes was found compared to the time after three implants were loaded with locator abutments. The dispersion of the values increased with the number of mastication cycles. The slopes increased significantly with the number of chewing cycles. For 10 versus 5 chewing cycles, a *p* value of 0.0024 was observed, and for 20 to 60 versus 5 chewing cycles, the *p* value was less than 0.0001. Mixing also increased with 5 chewing cycles (*p* value less than 0.0001). Preoperative chewing group, mixing significantly increased from 20 cycles and more chewing cycles than from 5 chewing cycles (*p* = 0.0001 and *p* < 0.0001, respectively)."

The average values of the mixture in percentage are shown in Table 1, which provides predicted median values for mixing in the mastication cycles used from the initial state (pre-op) to 1-year follow-up.

### Analysis of OHIP-G14 and sum score

**Fig. 7:**
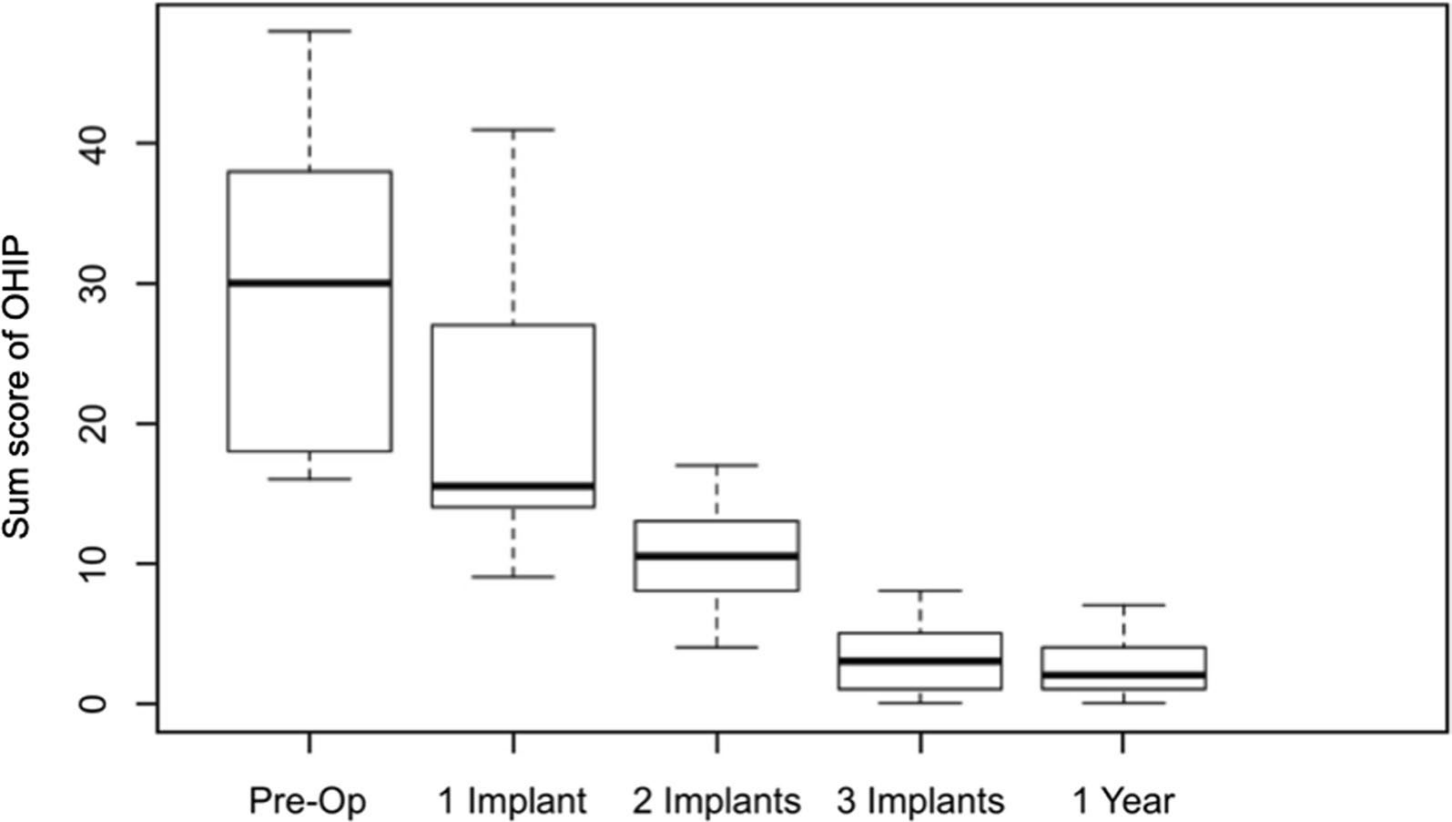
OHIP-G14 box-plot analysis: the mean OHIP-G14 score was 30.4 preoperatively, 21.1 after the activation of the first locator, 10.7 after the activation of two locator abutments, and 3.2 after the activation of all three locator abutments. After 1 year, there was no significant change in the OHIP-G14 score. The median score was 2.0, and the mean score was 2.6

The results of oral health-related quality of life are presented in box-plot analysis (Fig. 7), which shows a continuous decrease in the values. After the loading of three implants with locator abutments, the values had hardly changed. The dispersion of the values decreased with observed time which was verified in a mixed model. The mean value of OHIP-G14 was 30.4 before operation, 21.1 after loading the first implant, 10.7 after loading of other two implants, and 3.2 after loading all 3 implants. After 1 year, OHIP-G14 was 2.6 which implies no significant change. The result was checked in a similar mixed model. A weak decrease of 0.4 OHIP-G14 units per time point was estimated. No significance was detectable compared to the time of using of all three locator setups (*p* = 0.22).

**Fig. 8:**
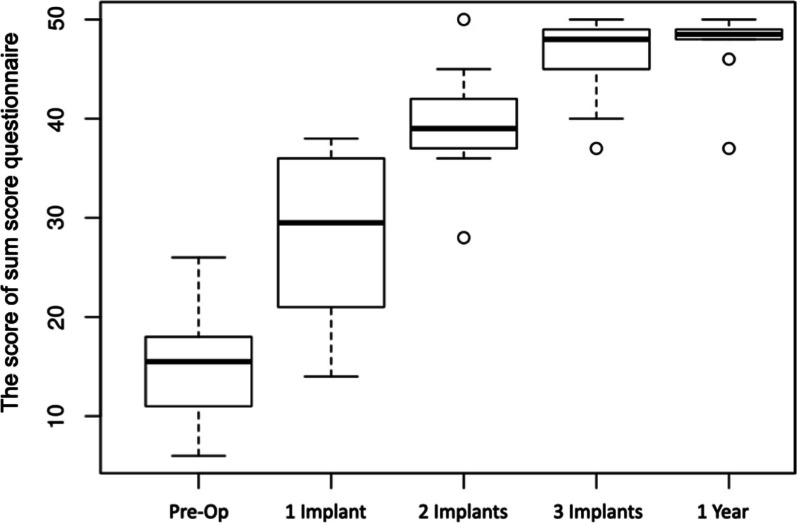
Sum score box-plot analysis: the mean value of the sum score was 15.5 preoperatively, 27.8 after the activation of the first locator, 39.4 after the activation of two locator abutments, and 46.2 after the activation of all three locator abutments. After 1 year, there was no significant change in the total score. The median score was then 48.5, and the mean score was 47.3

The mean of the sum score was 15.5 preoperatively, 27.8 after the first locator used, 39.4 after other two locators used, 46.2 after all three locators used, and 47.3 after 1 year (Fig. 8). The result was verified in a like mixed model. A weak increase of 0.7 sum score units per time point was estimated. No significance was detectable, analogous to OHIP-G14, compared to the time of loading all three implants (*p* = 0.22).

The quality of life and ME depend greatly on the time point, from preoperative to the activation of all three locator abutments. This implies a correlation between the quality of life and ME, as shown by a sequential analysis of variance. In this analysis, the dependence was first evaluated based only on ME, and then additionally on the time point (i.e., the number of locator abutments). When the time point is fixed, ME has no discernible influence on the quality of life (coefficient of 0.11, *p* = 0.67). However, when ME is the same at different time points, the timing (i.e., the number of locator setups) has a significant effect on the quality of life (coefficient of − 8.57, *p* < 0.001). Without considering time, quality of life significantly correlates with ME.

## Discussion

Masticatory performance can be objectively evaluated using various methods, including bite force measurement, food mastication sieving, color-changing gum assessment, recording jaw mechanics, and muscle activity. The time or number of chewing strokes is often employed to represent masticatory performance. In this study, it was concluded that the two-color chewing gum test is a reliable method for assessing masticatory performance in complete denture wearers. This assessment utilizes both visual and electronic calorimetric analyses, as mentioned in previous publications [23, 24]. The validation of this method was further established by testing the two-color chewing gum for chewing cycles in conjunction with maximum bite force measurements. The results indicate that digital image processing of the two-color chewing gum test specimens provides reliable quantitative data for assessing chewing efficiency.

Masticatory function test by utilizing chewing gum is an ideal examination for geriatric patients whom wear overdenture. Unlike other food, chewing gum simulates the natural chewing process and not able to be swallowed; therefore, no sample will lost in the study analysis [25]. On the other hand, geriatric patients often suffer from xerostomia. It can be unpleasant for patients to chew the test food when the saliva is insufficient and affects the result from questionnaires [26]. According to Liedberg et al., the salivary flow rate does not influence the results of the chewing gum test, making it suitable for patients with xerostomia [27]. The feasibility of using Mentos Fruit 3 as a chewing gum test had already been verified in Ludwig's previous dissertation [20]. The results demonstrated that selecting green and red Mentos gum produced reliable and reproducible quantitative data in different chewing cycle.

It has been shown that the evaluation the color changes of chewing gum can be applied as a tool to investigate ME [24–27]. In fact, it has already been successfully tested with the same method in a previous study with full-mouth prosthesis [28, 29], in which it shown that an increase in the number of chewing cycles is accompanied by an increase in color mixing. Furthermore, implants supported fixed restoration in the mandible achieved a higher rate of color mixing compared to the conventional removable denture. In a short-term randomized trial, the study assessed chewing efficiency in conventional dentures, fixed prostheses, and milled bar overdentures used for All-on-4 implant rehabilitation of atrophied mandibular ridges. Milled bar overdentures were found to be associated with significantly higher chewing efficiency compared to fixed prostheses. The blending indices in the overdenture group were significantly better, consistent with the results of the present study [30].

A continuous increase in MF was observed from the period of first implant loading to the three implants. In the point of 1-year follow-up, the MF changed was not significant and remained at a relatively level. These results suggest that MF increases with the medium implant, but clear improvement is observed with using at least two implants. It could be explained by the fact that the distribution of two implants provided better support with the attachments, which a single implant could not achieve. However, a range of complex motions in multiple axes of rotation still posed a challenge with two implants. With the use of three implants, there was a clear increase in stability due to the polygonal distribution. Therefore, an overdenture with triangular support using three implants had greater stability than one or two implants, contributing to greater masticatory efficiency.

Ludwig's research reported that mandibular prostheses with implant support have twice the masticatory efficiency compared to removable prostheses [20]. In addition, other studies have also shown that the masticatory force of implant-retained overdentures is more efficient than that of completely conventional dentures [31–33]. Although ME depends primarily on the function and the condition of remaining teeth and the type of restoration [32]. Other factors such as reduced salivary flow or muscle strength also resulting in reducing ME [34]. According to Carlsson et al., the occlusal pattern is more crucial than the number of teeth in estimating ME [35]. However, due to the small cases amount, these parameters were not determined in the present study. Nevertheless, result demonstrates a clear correlation between the number of implants and ME. With a small chewing cycles, less significant results were obtained because the overall mixing was relatively low. On the contrast, with a high number of chewing cycles, the gums were highly mixed that differences were shown between groups. Schimmel et al. suggested to use 20 chewing cycles for comparing ME as a standard examination to simulate real chewing situation [29]. In other studies, the number of 20 chewing cycles was also given as a reference since the significant differences can be observed [36–39].

The results of questionnaire scores were correlated with significant improvement in masticatory performance. All patients responded with a relatively high OHIP-G14 score before operation. This indicated an unsatisfaction of masticatory function from the edentulous patients. After one implant loading, the decreased OHIP-G14 score was observed. Although, with utilization of two and three implants for the overdenture, the OHRQoL increased significantly, after 1-year follow, patients satisfied with their restoration without significant changed in OHIP-G14 score. The similar results also presented in sum score with ascending. The different from OHIP-G14, high values suggested interpreted positively with patients’ feedback. Study by Walton et al. reported different conclusions. Total denture wearers had one or two implants placed in the edentulous mandible [40]. No significant differences in satisfaction between patients were found by placing one or two implants retained overdenture in the edentulous mandible. However, the study only compared inter-individual performance and satisfaction. In a recent systematic review, 28 studies reporting patient-reported outcomes from 1457 patients were included. It conducted there was a significantly positive effect of an additional implant in mandibular implant overdenture [41].

The limitation of the present study is the relatively short follow-up time and lack of comprehensive analysis of restoration’s efficiency. A recent systematic review indicated a significant improvement in masticatory performance for implant-supported prostheses at 12–36 months after prosthesis delivery [42]. Future studies should prioritize investigating the long-term outcomes of overdenture efficiency, including aspects such as the loosening or repair of locator components, particularly with reasonable numbers of implants utilized in larger sample size.

## Conclusions

Given the limitation of a small number of cases and a short follow-up period, the present study's conclusions suggest that as the number of implants increases in the mandible, the masticatory efficiency consistently shows a significant increase, as demonstrated by the chewing gum test. With the investigation of the OHIP-G14 and sum score, the results of patient report outcome might be associated with the increase in the number of implants. Future studies need to be set up with a comparison group.

## Abbreviations

ISQ: Implant stability coefficients
ME: Masticatory efficiency
MF: Masticatory function
OHIP: Oral health impact profile
OHRQoL: Oral Health-related Quality of Life

## References

[1] Emami E, de Souza RF, Kabawat M, Feine JS (2013). The impact of edentulism on oral and general health. Int J Dent.

[2] Pietrokovski J, Starinsky R, Arensburg B, Kaffe I (2007). Morphologic characteristics of bony edentulous jaws. J Prosthodont.

[3] Tallgren A (2003). The continuing reduction of the residual alveolar ridges in complete denture wearers: a mixed-longitudinal study covering 25 years. 1972. J Prosthet Dent.

[4] Huumonen S, Haikola B, Oikarinen K, Soderholm AL, Remes-Lyly T, Sipila K (2012). Residual ridge resorption, lower denture stability and subjective complaints among edentulous individuals. J Oral Rehabil.

[5] Fiske J, Davis DM, Frances C, Gelbier S (1998). The emotional effects of tooth loss in edentulous people. Br Dent J.

[6] Awad MA, Locker D, Korner-Bitensky N, Feine JS (2000). Measuring the effect of intra-oral implant rehabilitation on health-related quality of life in a randomized controlled clinical trial. J Dent Res.

[7] Critchlow SB, Ellis JS (2010). Prognostic indicators for conventional complete denture therapy: a review of the literature. J Dent.

[8] Lahoud T, Yu AY, King S (2023). Masticatory dysfunction in older adults: a scoping review. J Oral Rehabil.

[9] Kutkut A, Bertoli E, Frazer R, Pinto-Sinai G, Fuentealba Hidalgo R, Studts J (2018). A systematic review of studies comparing conventional complete denture and implant retained overdenture. J Prosthodont Res.

[10] McGrath C, Comfort MB, Lo EC, Luo Y (2003). Patient-centred outcome measures in oral surgery: validity and sensitivity. Br J Oral Maxillofac Surg.

[11] Lam WY, McGrath CP, Botelho MG (2014). Impact of complications of single tooth restorations on oral health-related quality of life. Clin Oral Implants Res.

[12] Thomason JM, Kelly SA, Bendkowski A, Ellis JS (2012). Two implant retained overdentures—a review of the literature supporting the McGill and York consensus statements. J Dent.

[13] Karbach J, Hartmann S, Jahn-Eimermacher A, Wagner W (2015). Oral health-related quality of life in edentulous patients with two- vs four-locator-retained mandibular overdentures: a prospective, randomized, crossover study. Int J Oral Maxillofac Implants.

[14] Zitzmann NU, Marinello CP (2006). Patient satisfaction with removable implant-supported prostheses in the edentulous mandible. Schweiz Monatsschr Zahnmed.

[15] Padmanabhan H, Kumar SM, Kumar VA (2020). single implant retained overdenture treatment protocol: a systematic review and meta-analysis. J Prosthodont.

[16] Miler A, Correia ARM, Rocha JMC, Campos JCR, da Silva M (2017). Locator (R) attachment system for implant overdentures: a systematic review. Stomatologija.

[17] Schimmel M, Araujo M, Abou-Ayash S, Buser R, Ebenezer S, Fonseca M, Heitz-Mayfield LJ, Holtzman LP, Kamnoedboon P, Levine R, McKenna G, Maniewicz S, Matarazzo F, Mattheos N, Papaspyridakos P, De Souza AB, Srinivasan M, Stilwell C, Weber HP (2023). Group 4 ITI consensus report: patient benefits following implant treatment in partially and fully edentulous patients. Clin Oral Implants Res.

[18] Ayna M, Sagheb K, Gutwald R, Wieker H, Florke C, Acil Y (2020). A clinical study on the 6-year outcomes of immediately loaded three implants for completely edentulous mandibles: "the all-on-3 concept". Odontology.

[19] Aghaloo T, Pi-Anfruns J, Moshaverinia A, Sim D, Grogan T, Hadaya D (2019). The effects of systemic diseases and medications on implant osseointegration: a systematic review. Int J Oral Maxillofac Implants.

[20] Ludwigs LM. Vergleichende Untersuchung der mundgesundheitsbezogenen Lebensqualität und Kaueffizienz von Vollprothesenträgern mit und ohne implantologischer Retention im Unterkiefer [dissertation on the Internet]. Mainz: Johannes Gutemberg-Universität; 2018. https://bibliographie.ub.uni-mainz.de/catalog/publication-93391. Accessed 10 Oct 2023

[21] Edlund J, Lamm CJ (1980). Masticatory efficiency. J Oral Rehabil.

[22] Cawood JI, Howell RA (1988). A classification of the edentulous jaws. Int J Oral Maxillofac Surg.

[23] Silva LC, Nogueira TE, Rios LF, Schimmel M, Leles CR (2018). Reliability of a two-colour chewing gum test to assess masticatory performance in complete denture wearers. J Oral Rehabil.

[24] Vaccaro G, Pelaez JI, Gil JA (2016). Choosing the best image processing method for masticatory performance assessment when using two-coloured specimens. J Oral Rehabil.

[25] Hama Y, Kanazawa M, Minakuchi S, Uchida T, Sasaki Y (2014). Reliability and validity of a quantitative color scale to evaluate masticatory performance using color-changeable chewing gum. J Med Dent Sci.

[26] Kubota C, Kanazawa M, Hama Y, Komagamine Y, Minakuchi S (2017). Association between chewing-stimulated salivary flow under the effects of atropine and mixing ability assessed using a color-changeable chewing gum. J Prosthodont Res.

[27] Liedberg B, Owall B (1991). Masticatory ability in experimentally induced xerostomia. Dysphagia.

[28] Prinz JF (1999). Quantitative evaluation of the effect of bolus size and number of chewing strokes on the intra-oral mixing of a two-colour chewing gum. J Oral Rehabil.

[29] Schimmel M, Christou P, Herrmann F, Muller F (2007). A two-colour chewing gum test for masticatory efficiency: development of different assessment methods. J Oral Rehabil.

[30] Elsyad MA, Tella EAES, Mohamed SS, Mahrous AI (2022). Within-patient evaluation of chewing efficiency and maximum bite force of conventional dentures, fixed prostheses, and milled bar overdentures used for All-on-4 implant rehabilitation of atrophied mandibular ridges: a short-term randomized trial. Clin Implant Dent Relat Res.

[31] Fontijn-Tekamp FA, Slagter AP, Van Der Bilt A, Van THMA, Witter DJ, Kalk W (2000). Biting and chewing in overdentures, full dentures, and natural dentitions. J Dent Res.

[32] Burns DR (2000). Mandibular implant overdenture treatment: consensus and controversy. J Prosthodont.

[33] Chen L, Xie Q, Feng H, Lin Y, Li J (2002). The masticatory efficiency of mandibular implant-supported overdentures as compared with tooth-supported overdentures and complete dentures. J Oral Implantol.

[34] Müller F, Nitschke I (2005). Mundgesundheit, zahnstatus und ernährung im alter. Z Gerontol Geriatr.

[35] Carlsson GE (1974). Bite force and chewing efficiency. Front Oral Physiol.

[36] Hayakawa I, Watanabe I, Hirano S, Nagao M, Seki T (1998). A simple method for evaluating masticatory performance using a color-changeable chewing gum. Int J Prosthodont.

[37] Liedberg B, Owall B (1995). Oral bolus kneading and shaping measured with chewing gum. Dysphagia.

[38] Hirano K, Hirano S, Hayakawa I (2004). The role of oral sensorimotor function in masticatory ability. J Oral Rehabil.

[39] Anastassiadou V, Heath MR (2001). The development of a simple objective test of mastication suitable for older people, using chewing gums. Gerodontology.

[40] Walton JN, Glick N, Macentee MI (2009). A randomized clinical trial comparing patient satisfaction and prosthetic outcomes with mandibular overdentures retained by one or two implants. Int J Prosthodont.

[41] Abou-Ayash S, Fonseca M, Pieralli S, Reissmann DR (2023). Treatment effect of implant-supported fixed complete dentures and implant overdentures on patient-reported outcomes: a systematic review and meta-analysis. Clin Oral Implants Res.

[42] Srinivasan M, Kamnoedboon P, Angst L, Müller F (2023). Oral function in completely edentulous patients rehabilitated with implant-supported dental prostheses: a systematic review and meta-analysis. Clin Oral Implants Res.

